# An eight-year asymptomatic retention of a knife blade in the chest: a case report

**DOI:** 10.1093/jscr/rjaf325

**Published:** 2025-05-31

**Authors:** Nashivai Kivuyo, Alfred Chibwae, Goodluck Sanga, Arnold Matemu, Rajabu Bakari, Mwita Masire, Christian Issangya, Daniel Kitua, Frederick Lyimo, Maurice Mavura

**Affiliations:** Department of Surgery, Muhimbili University of Health and Allied Sciences, PO Box 65001, Dar es Salaam, Tanzania; Department of General Surgery, Muhimbili National Hospital, PO Box 65000, Dar es salaam, Tanzania; Department of Surgery, Muhimbili University of Health and Allied Sciences, PO Box 65001, Dar es Salaam, Tanzania; Department of Surgery, Muhimbili University of Health and Allied Sciences, PO Box 65001, Dar es Salaam, Tanzania; Department of Surgery, Muhimbili University of Health and Allied Sciences, PO Box 65001, Dar es Salaam, Tanzania; Department of Surgery, Muhimbili University of Health and Allied Sciences, PO Box 65001, Dar es Salaam, Tanzania; Department of General Surgery, Muhimbili National Hospital, PO Box 65000, Dar es salaam, Tanzania; The College of Surgeons of East, Central and Southern Africa, PO Box 1009, Arusha, Tanzania; Department of Surgery, Muhimbili University of Health and Allied Sciences, PO Box 65001, Dar es Salaam, Tanzania; Department of Surgery, Muhimbili University of Health and Allied Sciences, PO Box 65001, Dar es Salaam, Tanzania; Department of General Surgery, Muhimbili National Hospital, PO Box 65000, Dar es salaam, Tanzania; Department of Radiology and Diagnostic Imaging, Muhimbili National Hospital, PO Box 65000, Dar es Salaam, Tanzania; Department of General Surgery, Muhimbili National Hospital, PO Box 65000, Dar es salaam, Tanzania; The College of Surgeons of East, Central and Southern Africa, PO Box 1009, Arusha, Tanzania

**Keywords:** penetrating injury, retained foreign body, thoracotomy, thoracic surgery, trauma surgery

## Abstract

Retained foreign bodies in the thoracic cavity following penetrating trauma are rare and typically identified shortly after injury. However, prolonged asymptomatic retention is possible, posing diagnostic challenges especially in resource limited settings. A 44-year-old male, otherwise healthy, presented with a right chest wall sinus discharging pus, 8 years after sustaining multiple stab wounds treated with primary first aid only. Imaging performed during the current presentation revealed a retained knife blade within the right hemithorax. A right thoracotomy was performed, and the foreign body was successfully removed. The patient had an uncomplicated postoperative recovery. This case underscores the potential for significant delayed complications from untreated penetrating chest trauma, particularly in settings with limited access to definitive surgical care. Improved trauma management protocols, including enhanced initial evaluation and follow-up, are essential to prevent such long-term morbidity in resource-constrained environments.

## Introduction

Retained foreign bodies in the chest following penetrating trauma are a documented phenomenon, primarily resulting from ballistic injuries [1, 2]. Most literature focuses on small foreign bodies, such as bullets, which are often left behind due to their difficulty localizing and retrieving during emergency procedures [1, 3]. In developing countries where firearm use is uncommon, such injuries are also seldom encountered. In contrast, stab wounds represent the most frequent penetrating civilian injuries in such settings [4].

Few cases of retained large foreign bodies in the thoracic cavity following stab injuries have been reported in the literature, with most diagnoses occurring within weeks to months [4, 5]. However, we report a rare case of a retained knife blade in the thoracic cavity for eight years.

## Case presentations

### Clinical presentation

A 44-year-old male Tanzanian presented to our facility with a 10-day history of pus discharge below the right nipple. He denied any chest pain, difficulty breathing, cough, or fever. The patient recalled being involved in a violent altercation 8 years ago, during which he sustained multiple cuts to his face, back, chest, and abdomen. Following the incident, he sought first aid at a primary health facility, where his wounds were sutured; however, no radiological investigation was conducted at that time as hemostasis was achieved, despite of no suspicious of foreign body retention in part injured, there was no facilities for radiological investigations. He had an uneventful course over the next 8 years until his current presentation.

**Figure 1:**
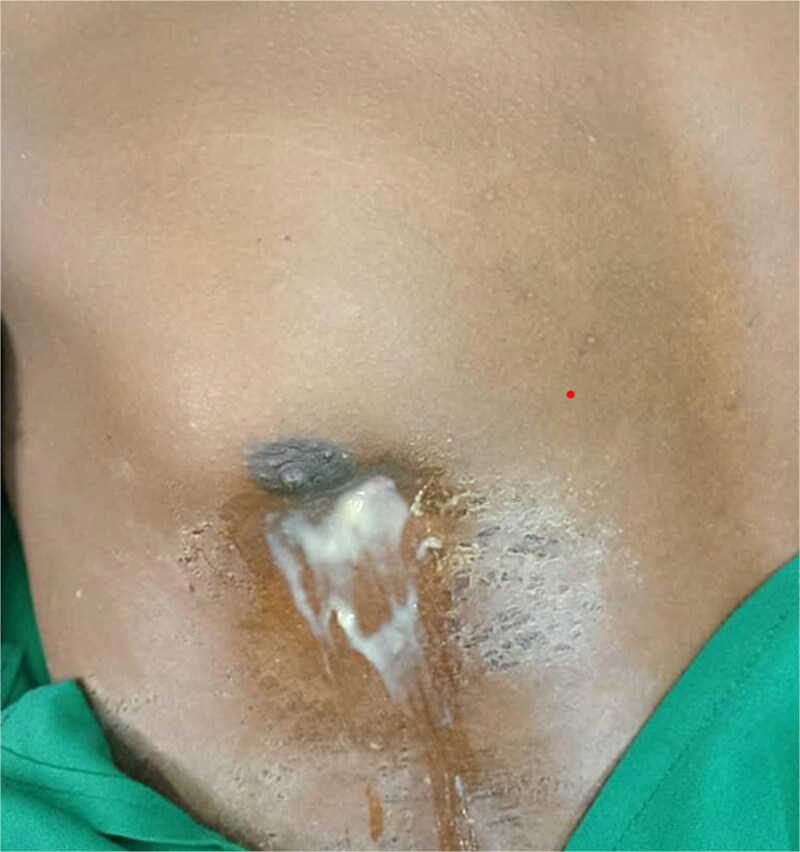
An image showing a pus-discharging sinus below the right nipple.

Upon examination, the patient’s vital signs were within normal limits. He had a relatively flattened right anterior rib cage compared to the left, with decreased chest expansion. Notably, a pus-discharging sinus was observed just below the right nipple, discharging foul-smelling pus with surrounding induration (Fig. 1).

### Radiological investigations

**Figure 2:**
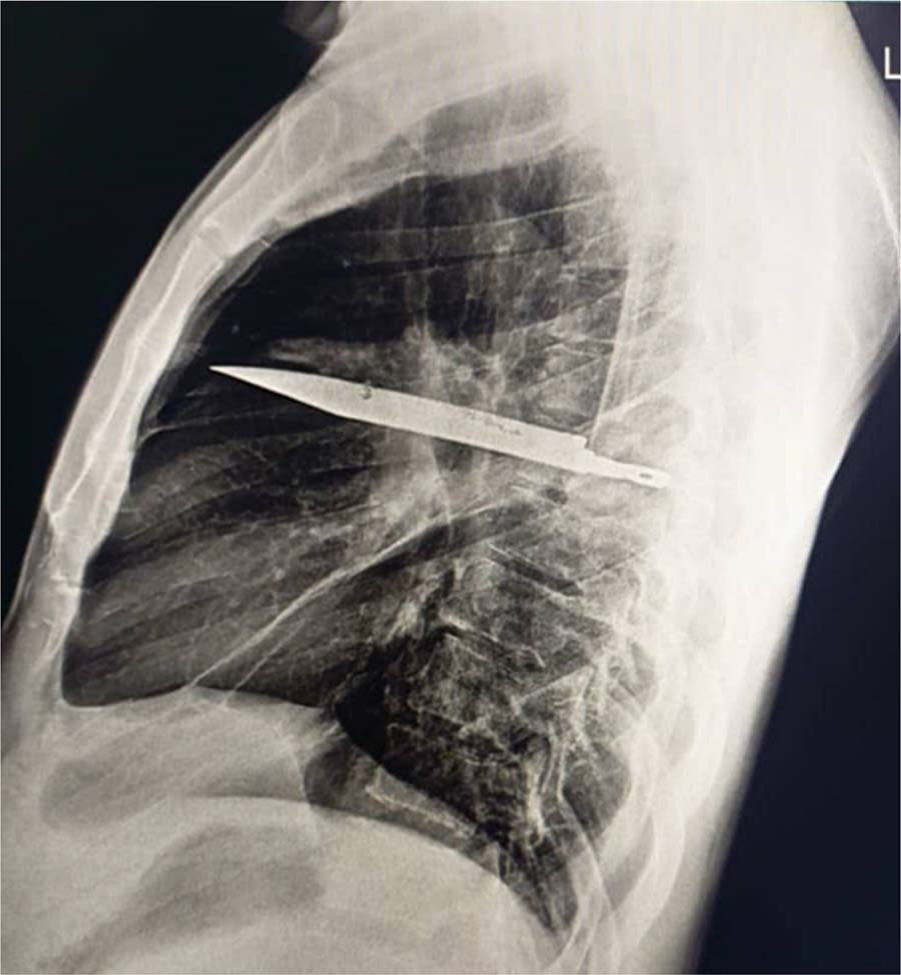
Lateral chest radiograph demonstrating a retained metallic object (knife) in the mid-thorax with surrounding opacification consistent with post-traumatic fibrosis following a stab wound.

**Figure 3:**
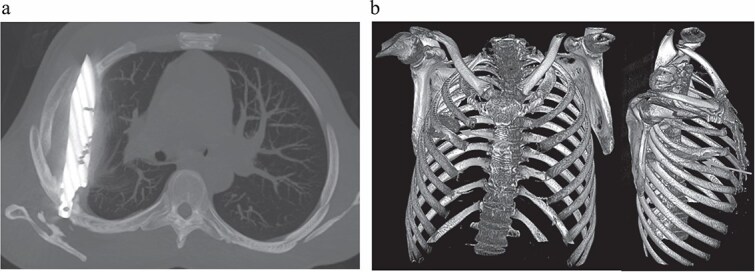
(a) CT chest maximum intensity projection (MIP) demonstrating a retained metallic foreign body (knife) traversing the right chest, with associated healed fractures. (b) 3D reconstructed CT chest image demonstrating the retained knife and associated skeletal injuries.

Initial imaging with a lateral chest radiograph demonstrated a retained metallic object in the mid-thorax, with surrounding opacification likely representing a resolving or chronic loculated hematoma or post-traumatic fibrosis, a sequela of the patient's stab wound (Fig. 2). Subsequent computed tomography (CT) imaging (Fig. 3a and b) revealed a retained foreign metallic object traversing the right chest. The entry point was identified through the right scapula, between the 5th and 6th posterior intercostal spaces, with the tip extending to the 3rd and 4th anterior intercostal spaces. CT also demonstrated healed fractures of the right scapula, 5th and 6th ribs posteriorly, and the 3rd rib anteriorly. An elliptical area of consolidation with central hypodensity was observed, further supporting the interpretation of a resolved or chronic loculated hematoma. The remainder of the lung parenchyma appeared unremarkable.

### Surgical management

**Figure 4:**
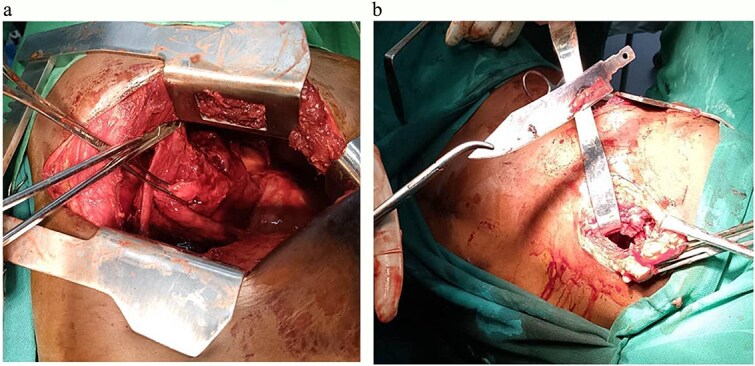
(a) An image showing a defect in middle lobe of the right lung after the removal of the large metallic foreign body. (b) An image showing a large metallic (knife) foreign body removed from the middle lobe of the right lung.

Thoracotomy via a posterolateral incision in the 5th intercostal space was done. Intraoperatively, multiple adhesions were encountered. A retained metallic knife blade was identified within the middle lobe, surrounded by pus and necrotic tissue. Adhesiolysis was performed with meticulous dissection to separate the lung lobes. The foreign body was carefully extracted, followed by pus drainage for culture and necrosectomy. The thoracic cavity was thoroughly irrigated with a sodium chloride solution, and an underwater seal drainage chest tube was placed under direct vision before wound closure (Fig. 4a and b). Patient was empirically kept on the broad spectrum awaiting for pus culture results which then reported to be negative after day seven.

### Post-operative care and follow-up

**Figure 5:**
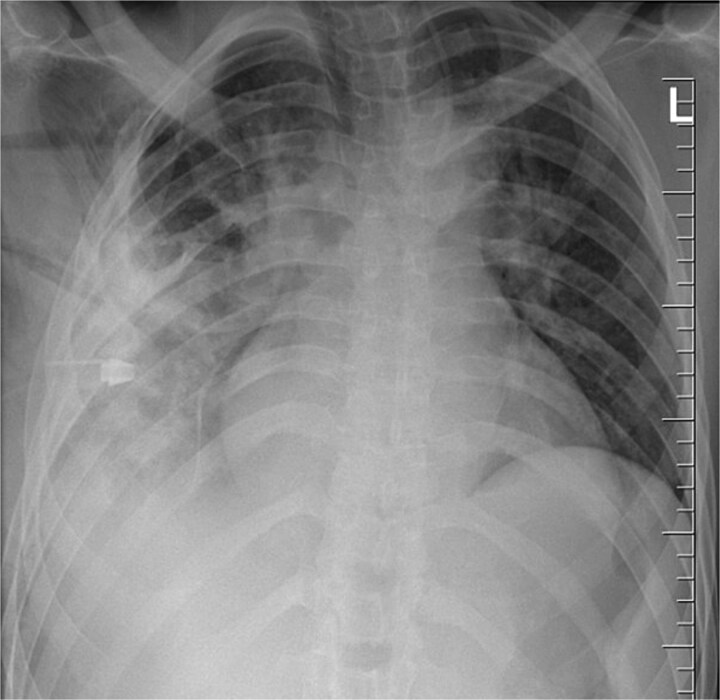
Chest radiograph post-operatively, demonstrating a chest tube in situ, residual lung opacification, and expected post-surgical changes following removal of a retained thoracic foreign body (knife).

The patient was monitored in the Surgical Intensive Care Unit for 24 hours before being transferred to a general ward. Seven days post-op, a chest X-ray showed a chest tube in situ and lung opacification, consistent with the pleural collection and expected changes (Fig. 5). The chest tube was removed on day eight when the drainage was less than 100 ml in 24 hrs., and the patient was discharged on postoperative day 10. His follow-up appointments on day 14 and week six were uneventful.

## Discussion

The current case report underscores the significant challenges in trauma care within resource-limited settings, where access to basic imaging and surgical expertise is limited [6–8]. After sustaining serious stab wounds eight years prior, the patient received only first aid, despite the need for imaging and subsequent surgery. While this patient recovered well after surgery, there was a considerable risk that the retained knife could lead to a fatal outcome [3].

Large retained intrapulmonary foreign bodies are associated with complications such as inflammation, tissue necrosis, and abscess formation, as seen in our patient [3–5]. However, our patient survived for eight years without experiencing any overt symptoms. The prolonged asymptomatic period likely resulted from the body's ability to encapsulate the foreign body within a fibrous capsule, limiting inflammation and tissue damage. This mechanism, seen in similar cases, helps contain foreign materials and delay clinical manifestations [9, 10]. However, this adaptive response can conceal underlying pathology, ultimately resulting in serious complications, as evidenced by the patient's presentation [9].

Managing these patients requires a multifaceted approach, starting with appropriate imaging studies like plain X-rays and CT scans to accurately identify the foreign body and evaluate the associated pathologies [11]. The effective surgical intervention involves removing the foreign body, pus drainage, and debridement as performed in our case [11]. Postoperative ICU monitoring allows for early detection and management of potential complications.

Although this patient fared well after surgery, there was a significant risk that the retained knife could have led to a fatal outcome [3]. Henceforth, this case illustrates the urgent need for increased awareness and improved protocols for trauma management safe surgery and safe anaesthesia in low-resource settings, prioritizing such as imaging studies and standardized guidelines for the evaluation and follow-up of trauma patients [12]. Additionally, fostering regular training opportunities for healthcare providers to enhance their ability to recognize the potential long-term complications associated with penetrating injuries.

In areas with limited resources, patients should be promptly referred to higher-level healthcare facilities capable of providing comprehensive diagnostic and surgical care [7, 8]. This will ensure that affected individuals receive timely and appropriate interventions, ultimately reducing the risk of complications, improving overall health outcomes, and enhancing the effectiveness of trauma care systems in these vulnerable communities.

## Data Availability

Not applicable for this case report.
